# Long-Term Results of Autologous Auricular Cartilage Graft Applied in Anophthalmic Orbits Unable to Wear Prosthesis

**DOI:** 10.1155/2019/7197063

**Published:** 2019-04-09

**Authors:** Yi-Lin Liao, Shu-Ya Wu, Yueh-Ju Tsai

**Affiliations:** ^1^Department of Ophthalmology, Chang Gung Memorial Hospital, Linkou, Taiwan; ^2^College of Medicine, Chang Gung University, Taoyuan, Taiwan; ^3^Department of Ophthalmology, Taipei Tzu Chi Hospital, New Taipei City, Taiwan

## Abstract

In anophthalmic patients, shallow lower fornices make wearing ocular prostheses impossible and maintaining normal social activities difficult. This study retrospectively investigated the long-term surgical outcomes of autologous auricular cartilage grafting for contracted orbits. From 1995 to 2013, 29 anophthalmic contracture sockets with inadequate lower fornices and poor prosthesis retention presented to Chang Gung Memorial Hospital in Linkou, Taiwan, were treated using this surgical method. The success rate, aesthetic outcome, recurrence, and complications were analyzed. Among the 29 patients, 15 were women, 14 were men, their mean age was 45 years, and the mean follow-up time was 52 months (range = 6–159 months). Satisfactory lid position was achieved in 25 cases (86%), and lower fornix retraction recurred in four cases (14%). Neither donor site morbidity nor auricular deformity was noted during the follow-up period. Therefore, an auricular cartilage graft can be used successfully as a compatible spacer for anophthalmic patients with shallow lower fornices and prosthesis-fitting problems in long-term follow-up.

## 1. Introduction

Anophthalmic patients who wear an ocular prosthesis often experience orbital and eyelid abnormalities, such as enophthalmos, upper eyelid ptosis, deep superior sulcus, lower eyelid malposition, and shallow lower fornix. These symptoms constitute what is referred to as postenucleation socket syndrome (PESS) [1]. To manage these cosmetic disfigurements, multiple surgeries may be conducted in anophthalmic orbits. First, patients presenting with a volume deﬁcit undergo correction with a secondary implant (if no implant is present) or with an orbital ﬂoor implant (if a small intraconal implant is present) before any eyelid surgery [2]. In the past, lining deficits were corrected by ocular surface reconstructions and posterior lamella lengthening procedures with various spacers. However, using an orbital implant to correct enophthalmos may compromise the depth of the lower fornix if implant migration or protrusion occurs. Therefore, such patients might be unable to wear prostheses in daily activities and present to clinics with socket contracture.

Socket contracture ranges from mild-to-moderate and severe forms. Mild contracture refers to cicatricial entropion of the lower eyelids caused by fibrotic contracture in the posterior lamella. Cases whose contracture of the inferior fornices leads to the inability to maintain an ocular prosthesis are categorized as moderate socket contracture. Severe socket contracture manifests as marked vertical and horizontal forniceal contracture [3] and may occur following trauma, recurrent inflammation caused by an inadequate prosthesis, or radiotherapy.

An ongoing challenge for surgeons is developing a method to reconstruct moderate to severe contracture sockets with a lining deficit of the fornix, especially in non-Caucasian populations with prominent fibrosis tendency. Both scarring of the orbital fibrous connective tissue and disturbances to the conjunctiva contribute to socket contracture. Therefore, this study aims to report surgical outcomes for the use of autologous auricular cartilage grafting for moderate socket contracture in non-Caucasian populations as a reference for further studies.

## 2. Materials and Methods

We performed a retrospective review until 2017 of the medical records of patients who underwent fornix reconstruction with autologous auricular cartilage grafting by four surgeons at the Ophthalmic Plastic and Reconstructive Clinic of Linkou Chang Gung Memorial Hospital, Taiwan, from 1995 to 2013. The criteria for patient selection were anophthalmic patients with prosthesis-fitting problems, lower shallow fornices, and autologous auricular cartilage grafting. Posterior lamella lengthening surgery with auricular cartilage grafting was arranged after informed consent was obtained. The prostheses were made by the same ocularist. Patients with a follow-up period of less than 6 months and a history of lower fornix reconstruction were excluded. The study was approved by the Institutional Review Board of Chang Gung Medical University.

The causes of anophthalmic status, operation history, and lower lid malposition were investigated prior to surgery (see Table S1, the study database in Supplementary Materials). We classified patients into two groups: group 1 comprised those unable to wear prostheses and group 2 comprised those who wore prostheses with an upward-gaze pattern. Furthermore, we recorded the position of the prosthesis relative to the lower eyelid, supplemental lid surgery, any complications, and donor site morbidities after follow-up until the end of 2017.

**Figure 1:**
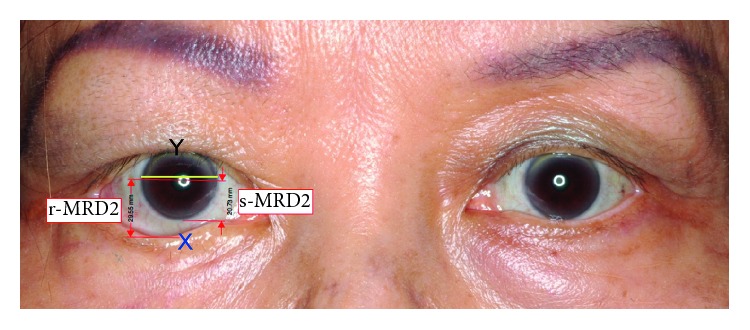
Lower lid real scleral show calculation.

The authors took preoperative and postoperative photos of the patients in sitting and primary gaze positions; they also measured the position of the lower eyelid in millimeters using Adobe Acrobat Pro IX (version 9.0.0; Adobe, San Jose, CA, USA) from the inferior limbus to the lower lid margin in each photo. To increase measurement accuracy, these photographs were uniformly adjusted for size ratios. The corneal horizontal diameter (*Y*), which is relatively constant in the eyes, was also measured. The reported mean corneal diameter among Chinese people is 12 mm [4]; the lower scleral show (*X*) was adjusted accordingly (Figure 1). Success was determined when the patient could wear an average-sized prosthesis without regrafting.

Scleral show (*X*) is the difference between real-MRD2 and standard-MRD2, and *Y* is the horizontal corneal diameter; real scleral show (mm) = *X*/*Y* × 12.

Statistical analysis was conducted using SPSS version 23.0 (SPSS, Chicago, IL, USA), and *p* < 0.05 was considered statistically significant. We used chi-square tests to compare the dislocation group with the upward-gaze group in terms of the necessity of additional lid procedures. Survival analysis was applied to the differences in surgical success between the two groups with the log-rank test.

## 3. Surgical Techniques

**Figure 2:**
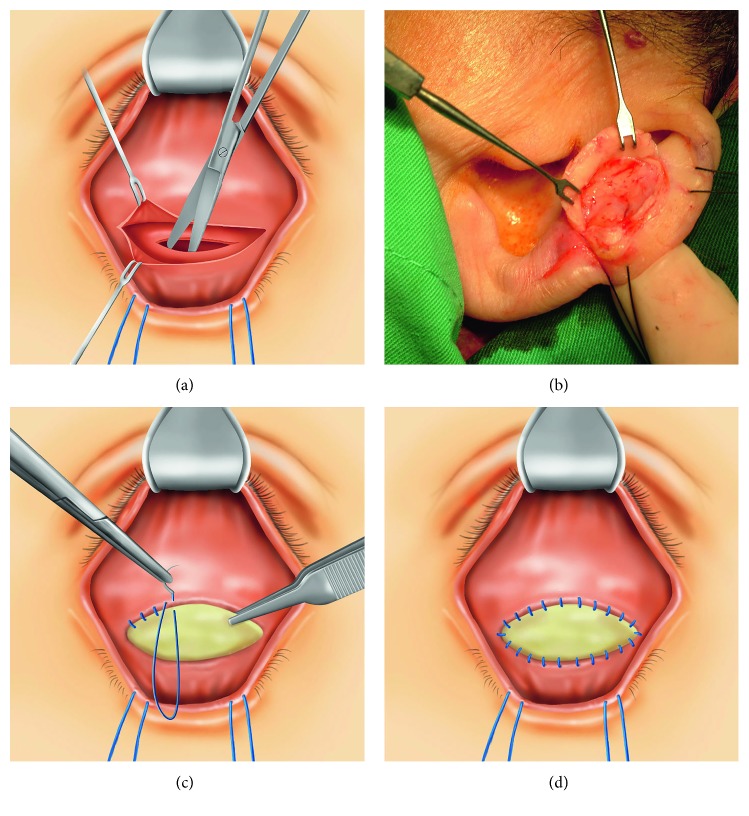
Surgical procedure of lower fornix reconstruction in anophthalmic sockets. (a) Making a conjunctival incision to release the adhesion of the cul-de-sac. (b) Harvesting the auricular cartilage graft from the scaphoid fossa. (c) Suturing the conjunctival edges to the auricular cartilage graft with a running 6-0 polyglactin 910 suture. (d) Cartilage grafting to restore the space of lower shallow fornix.

The shallow conjunctival cul-de-sac was infiltrated with 2% xylocaine and 1 : 200,000 adrenaline. A transconjunctival incision was made to release cicatricial adhesion of the lower lid (Figure 2(a)). After measuring the conjunctival gap, we harvested a cartilage graft from the scaphoid fossa of the donor ear. The skin and perichondrium were dissected to expose the anterior surface of the scaphoid fossa after infiltration of local anesthesia. Subsequently, an ideal auricular cartilage strip was incised and harvested (Figure 2(b)) [5]. The preauricular skin wound was closed with absorbable 5-0 polyglactin 910 interrupted sutures and compressed using a beta-iodine cotton ball to prevent hematoma after surgery. The cartilage graft was sutured between the tarsus and retractor with absorbable 6-0 polyglactin 910 continuous sutures (Figures 2(c) and 2(d)).

**Figure 3:**
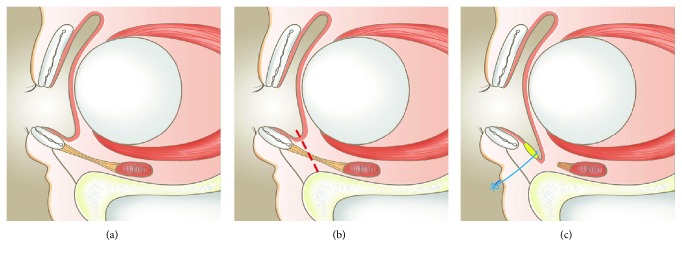
Sagittal view of lower fornix reconstruction with auricular cartilage graft. (a) Lower shallow fornix. (b) The dissection plane is represented by the dotted line. (c) The auricular cartilage graft was fixed to the anterior lamella, providing a proper space for wearing a prosthesis.

The graft was anchored to the anterior lamella of the lower lid with two absorbable 5-0 polyglactin 910 sutures (Figure 3), and a conformer was inserted to maintain the graft at a position proximal to the vascular bed and deepen the lower fornix. Next, the traction suture was made upward to the brow for 2 weeks during the rapid wound-healing phase. In our technique, the steps crucial to our surgical success were the anchoring procedures from posterior to anterior lamella, conformer insertion, and traction suture. Postoperative follow-up evaluations were performed at 2 weeks, 4 weeks, 3 months, and 6 months.

## 4. Results

In total, 29 anophthalmic patients (29 eyelids) underwent lower fornix reconstruction with autologous auricular cartilage grafting from 1995 to 2013. Patients' ages ranged from 10 to 87 years (mean = 45 years): 14 were men and 15 were women. All surgeries were performed under local anesthesia, except for those of two children. The mean length of postoperative follow-up was 52 months (ranging from 6 to 159 months).  Table 1 summarizes the demographic data of these patients as well as their anophthalmos etiologies. The most common etiology of anophthalmic status was trauma (69%), and others included malignancy (17%), congenital anomaly (10%), and endophthalmitis (4%).

In addition, we investigated the precipitating factors of shallow lower fornix, such as previous enophthalmos correction, radiation, chronic socket inflammation, orbital implant exposure, and orbital implant migration. The most common cause of the shallow lower fornix was previous enophthalmos correction with orbital floor implant (17%), and other causes were contracture sockets with history of implant exposure (7%) and irradiated sockets (7%).  Table 2 summarizes the prosthesis status and lower scleral show before and after surgery. In the dislocation group (group 1), the preoperative lower scleral show was unmeasurable because of loss of the lower fornix. After auricular cartilage grafting, the amount of lower scleral show was reduced to an average of 0.18 mm. In the upward-gaze prosthesis group (group 2), the amount of lower scleral show was reduced from 1.86 mm to 0.11 mm postoperatively. Thus, performing additional lid procedures such as lateral tarsal strip, horizontal shortening, and a full-thickness skin graft seemed more necessary in the dislocation group (55%) than in the upward-gaze group (22%).

**Figure 4:**
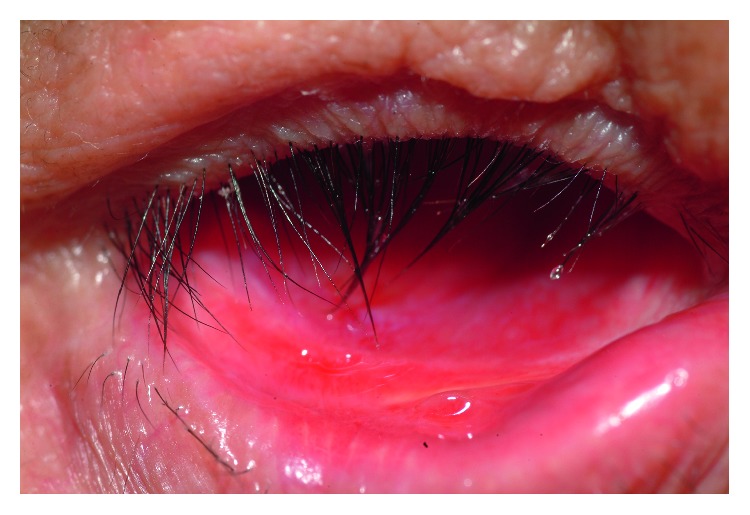
Auricular cartilage graft that took well after surgery.

**Figure 5:**
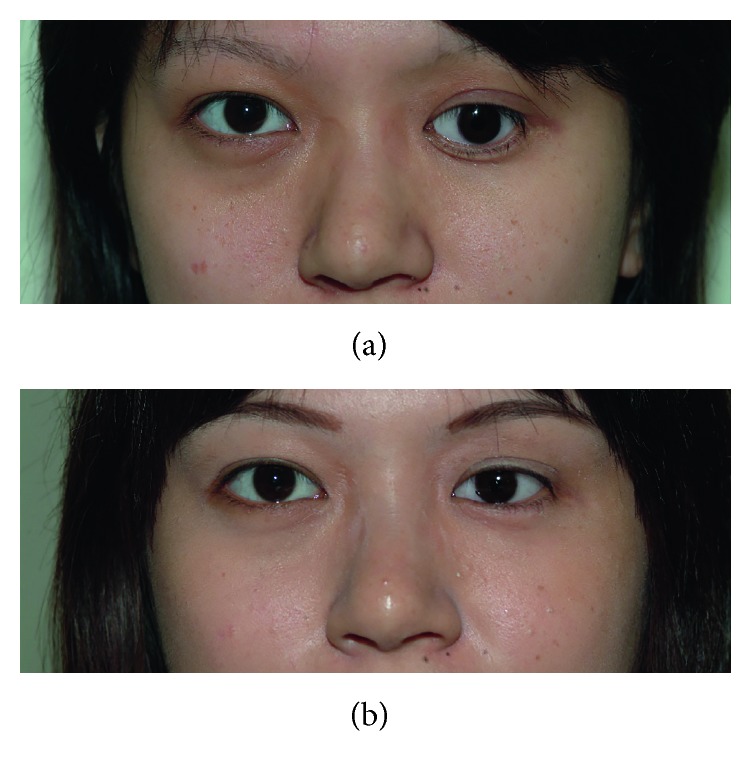
External photography before and after surgery. (a) 21-year-old woman had prosthesis dislocation on the left side. (b) Both prosthesis retention and symmetry of the eyes were improved 6 months after surgery.

Surgical success was defined as the graft taking well (Figure 4) and strong prosthesis retention after auricular cartilage grafting without a regraft procedure during follow-up. The difference in the success rate between the dislocation and upward-gaze groups was not statistically significant, with a mean follow-up of 52.45 ± 48.95 months (range = 6–159 months) (*p*=0.567). Thus, ocular prostheses can not only be maintained in an optimal position but also with a high level of symmetry to the other eye after lower fornix reconstruction with auricular cartilage grafting (Figure 5).

**Figure 6:**
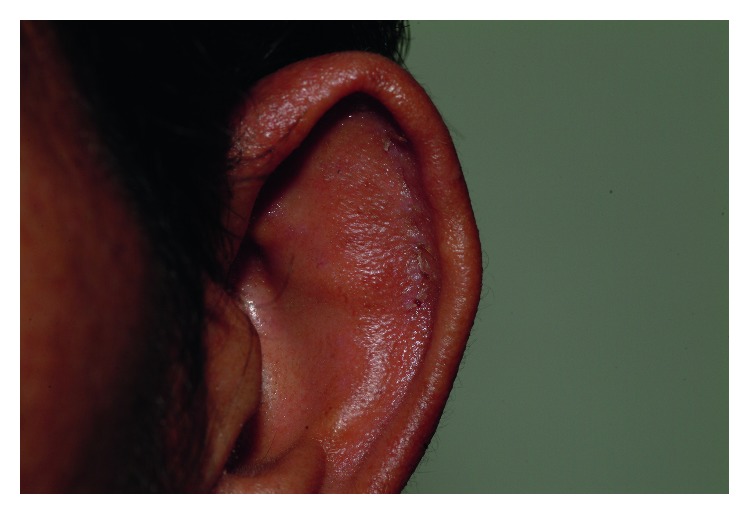
Close-up photo of an ear 2 weeks after cartilage grafting.

No donor site morbidity concerning the ears was reported during our long-term follow-up (Figure 6). Only minor complications, such as six lower fornix granulomas and one lower lid induration, were observed in the 29 cases. Most cases of pyogenic granuloma occurred 5–8 weeks after surgery and were easily treated with direct excision and topical mitomycin C.

## 5. Discussion

Lower fornix contracture in anophthalmic sockets often prevents patients from comfortably wearing ocular prostheses and affects their quality of life. To address this problem, many types of lower fornix reconstruction with or without grafts have been adopted. Sockets with minor contracture and only a short posterior lamella could be treated with marginal rotation and transverse blepharotomy. In sockets with moderate contracture, a shallow fornix, and inadequate conjunctival lining, mucous membrane grafts with silicone stents to the rim or conformers were used [6].

Furthermore, various spacers have been used to lengthen the posterior lamella of the lower lids; autologous grafts have included the hard palate mucosa, tarsus, oral mucosa membrane, dermis fat, radial forearm free flap [7–12], chondrocutaneous graft, and auricular cartilage graft [13–15]. Homologous grafts, such as amniotic membrane grafts, sclera, and fascia lata [16, 17], have also been applied as spacers to reconstruct retracted lower lids. Some surgeons have used synthetic spacer materials such as the acellular dermis graft and rigid, nylon, foil-anchored e-polytetrafluoroethylene stenting [18–20].

Thus far, no particular type of graft has been considered ideal or perfect for anophthalmic sockets; therefore, many types of grafts have been used as spacers for repairing lower lid retraction. Moreover, grafts have been used in repairing the lower lid retraction of sighted eyes. Some studies have explored the success rate of mucous membrane grafts in sighted eyes, but few have focused on the success rate of mucous membrane grafts for anophthalmic patients after long-term follow-up.

The success rate of hard palate grafts in displaced lower eyelids has ranged from 80% to 85% [7, 21]. In Holck's study, satisfactory lid position was achieved after hard palate grafting in 8 out of 10 anophthalmic sockets. Amniotic membrane grafting in 10 sockets with mild-to-moderate contracture provided comparable results to the oral mucosa grafting in Bajaj's 6-month follow-up study [10]. In 2008, Smith and Malet published a report stating that auricular cartilage grafting had a correction success rate of 92% among Caucasian anophthalmic patients who had lid retraction during an average follow-up of 19.7 months (range = 5–55 months) [15].

In the present study, the successful correction of 86% of 29 anophthalmic sockets was achieved through autologous auricular cartilage grafting after an average follow-up of 52.45 months. Thus, auricular cartilage, which possesses numerous beneficial characteristics, could serve as a lower lid spacer in non-Caucasian contracture sockets. The auricular cartilage harvested from the scaphoid fossa had a more appropriate curvature to fit ocular prostheses than did that from the concha area in the lower lid fornix. Moreover, it provided tailored sizes for individuals who required socket reconstruction. The surgical field was clear from the anterior approach, and harvesting auricular cartilage from the scaphoid fossa could be easily performed. After all adhesions at the shallow lower fornix were released, the auricular cartilage could offer an excellent scaffold for promoting conjunctiva re-epithelization.

Acellular dermis contracts significantly more hard palate mucosa when used as a lower eyelid spacer graft [18]. Compared with hard palate mucosa grafts, auricular cartilage grafts are more rigid and durable against contracture during the wound-healing process in non-Caucasian populations. They can also provide stable support for lower lids without absorption [22]. Furthermore, neither donor site morbidity nor functional change of ears has been reported so far. One study reported uncomplicated postoperative donor site bleeding in hard palate mucosa grafting [23], but this did not occur in our auricular cartilage grafts. Postoperative donor site care is simple, and patients' recovery time is short. A minor postoperative complication might be pyogenic granuloma within 2 months, which can be treated easily. Malignant changes after wearing prostheses for long term should be monitored if multiple recurrent episodes of ocular prosthesis dislocation occur following reconstruction. Because of the limitations of this retrospective study, the follow-up time varied from 6 to 159 months after medical records were reviewed. Thus, a further prospective study with a large series should be conducted to avoid loss to follow-up bias.

In conclusion, an auricular cartilage graft can be used successfully as a compatible spacer for non-Caucasian anophthalmic patients who exhibit shallow lower fornices and prosthesis-fitting problems at long-term follow-up. This graft offers not only lengthening of the posterior lamellae but also walling of the fornix to maintain a proper space for the prosthesis. It is a surgeon-friendly procedure for restoring patients' quality of life without cosmetic disfigurement or donor site morbidities.

## Figures and Tables

**Table 1:** Demographic characteristics of the anophthalmic patients in this study.

		No. of patients (%)	Mean	Range
Age	Years		44.69	10–87

Sex	Male	14 (48)		
	Female	15 (52)		

Etiology of anophthalmos	Trauma	20 (69)		
	Malignancy	5 (17)		
	Congenital anomaly	3 (10)		
	Endophthalmitis	1 (4)		

Follow-up time	Months		52.45	6–159

**Table 2:** Preoperative lower sclera show, additional lid surgery, and surgical outcomes.

		Group 1	Group 2	Overall
Prosthesis status		Dislocation	Upward-gaze	
No. of patients		11	18	
Lower sclera show (mm)	Pre-op	N/A	1.86 ± 0.90	
	Post-op	0.18 ± 0.60	0.11 ± 0.30	
Supplemental lid surgery	Lateral tarsal strip/FTSG	6/11 (55%)	4/18 (22%)	10/29 (34%)
Success rate		9/11 (82%)	16/18 (89%)	25/29 (86%)

N/A: unmeasurable.

## Data Availability

The statistics data used to support the findings of this study are included within the supplementary information file.

## Supplementary Materials

Supplementary Materials In supplementary table S1, we show the database of our clinical study. This provides additional information regarding past histories, previous surgeries, and postoperative complications.
